# Novel automated spinal ultrasound segmentation approach for scoliosis visualization

**DOI:** 10.3389/fphys.2022.1051808

**Published:** 2022-10-24

**Authors:** Weiwei Jiang, Fang Mei, Qiaolin Xie

**Affiliations:** College of Computer Science and Technology, Zhejiang University of Technology, Hangzhou, China

**Keywords:** ultrasound, medical image segmentation, scoliosis, 3D ultrasound image reconstruction, deep learning

## Abstract

Scoliosis is a 3D deformity of the spine in which one or more segments of the spine curve laterally, usually with rotation of the vertebral body. Generally, having a Cobb angle (Cobb) greater than 10° can be considered scoliosis. In spine imaging, reliable and accurate identification and segmentation of bony features are crucial for scoliosis assessment, disease diagnosis, and treatment planning. Compared with commonly used X-ray detection methods, ultrasound has received extensive attention from researchers in the past years because of its lack of radiation, high real-time performance, and low price. On the basis of our previous research on spinal ultrasound imaging, this work combines artificial intelligence methods to create a new spine ultrasound image segmentation model called ultrasound global guidance block network (UGBNet), which provides a completely automatic and reliable spine segmentation and scoliosis visualization approach. Our network incorporates a global guidance block module that integrates spatial and channel attention, through which long-range feature dependencies and contextual scale information are learned. We evaluate the performance of the proposed model in semantic segmentation on spinal ultrasound datasets through extensive experiments with several classical learning segmentation methods, such as UNet. Results show that our method performs better than other approaches. Our UGBNet significantly improves segmentation precision, which can reach 74.2% on the evaluation metric of the Dice score.

## 1 Introduction

Scoliosis is a 3D deformity of the spine, and it includes coronal, sagittal, and axial sequence abnormalities (Konieczny et al., 2013). The commonly used detection method today is to take a standing-position, full-spine X-ray. If the frontal X-ray film shows that the spine has a lateral curvature of more than 10°, the person is diagnosed as having scoliosis (Kawchuk and Mcarthur, 1997). The causes of scoliosis include congenital, acquired, or degenerative problems, but the cause of most scoliosis cases is unknown; this type is called idiopathic scoliosis, which is the most common type of scoliosis at present. According to statistics, about 80% of scoliosis cases belong to this category, and the difference between genders is significant, with women outnumbering men by 7:1. This condition often occurs during adolescence and is known as adolescent idiopathic scoliosis (AIS) (Weinstein et al., 2008). During the period of rapid growth, the development accelerates and then gradually deteriorates, leading to complications. For mild patients, conservative treatment with brace correction can be adopted (Negrini et al., 2011), but for severe patients, permanent spinal fusion surgery is a common method. However, permanent spinal fusion surgery greatly limits the patient’s range of motion, and the complexity of the surgery is prone to complications. Therefore, the best treatment is early detection and frequent monitoring (Reamy and Slakey, 2001).

Currently, the clinical detection of scoliosis mainly relies on X-rays. However, X-rays are radioactive, and routine monitoring during rehabilitation interventions is not advisable. In addition, observing the 3D structure of the human spine on X-ray films is difficult. Magnetic resonance imaging (MRI) is a safe imaging examination based on the principle of nuclear magnetic resonance. However, MRI usually requires the patient to be in a prone position, which may cause changes in spinous process morphology (Sailer et al., 2008). Moreover, it is expensive and takes a long time, so it cannot be used on occasions with high real-time requirements. Compared with the commonly used X-ray and MRI detection methods, ultrasound has no radiation, high real-time performance, and low price (Yang et al., 2021). Ultrasound is a mechanical wave generated by mechanical vibration (Ahmed et al., 2018). The wave can enter the human body and pass through various tissues to generate echoes and form images through computer calculations. Therefore, spine ultrasound imaging has become a research hotspot in the field of spine imaging.

2D images cannot be directly used to guide the examination of scoliosis due to the limitation of visual field space in 2D images. Recent studies have indicated that 3D ultrasound has a broad application prospect in the diagnosis of scoliosis. How to visualize the 3D shape of the spine and use it for subsequent clinical research has become a hot issue. Extended field-of-view ultrasound (United States EFOV) imaging is a technique used extensively in the clinical field to attain interpretable panorama of anatomy. Huang et alproposed a novel method called double-sweep 2.5-D EFOV to better image the spinal tissues and easily compute the Cobb angle (Huang et al., 2019). Cheung et aldeveloped a 3D ultrasound system to assess AIS (Cheung et al., 2013). A novel 2.5D extended field of view method was proposed for the assessment of scoliosis (Huang et al., 2018). Zheng et aldeveloped a 3D ultrasound imaging system called Scolioscan that can be used for spine scanning (Zheng et al., 2016). In recent years, with the gradual maturity of artificial intelligence theory and applications, deep learning technology has been widely used in the field of image processing (Cai et al., 2020). Huang et alproposed a new imaging method (Huang et al., 2022) to generate the 3D structure of the human spine through tracked freehand United States scanning. Tiny-YOLOv3 (Yi et al., 2019) and K-means clustering were applied in their study to predict the spatial location of vertebral landmarks; then, they modeled the vertebrae based on the spatial position of the vertebral landmarks to form the whole spine (Huang et al., 2022). Another state-of-the-art research method was proposed by Ungi et al. (2020). They used Unet (Ronneberger et al., 2015) to segment bony features in ultrasound images and realized the visualization of 3D spine models and measurement of scoliosis degree with the help of 3DSlicer.

Inspired by these previous studies, we adopted a two-stage processing strategy to visualize a 3D model of the spine. Different from Ungi et al., we developed a novel segmentation network for spinal ultrasound images, namely, ultrasound global guidance block network (UGBNet), to achieve an accurate segmentation of 2D spinal ultrasound images. Traditional convolution neural network segmentation, such as UNet, has local receptive fields, lacks long-term dependence, and is unable to make full use of the object-to-object relationship in the global view, which may lead to potential differences between the corresponding features of pixels with the same label. At the same time, these networks do not make full use of the feature information of the intermediate convolutional layer and ignore the global context information of different scales. In the current work, the proposed network UGBNet learns long-range feature dependencies through the global guidance block (GGB) module and aggregates non-local features in a spatial-wise and channel-wise manner after processing by the GGB module to obtain accurate segmentation results. The effective information obtained from the segmentation is combined with the position information obtained from the freehand ultrasound imaging system (Cheung et al., 2015) to visualize the 3D structure of the human spine. This inexpensive approach is convenient and intuitive in displaying the spine shape, realizes the visualization of the 3D shape of the spine, and is important for doctors’ follow-up diagnoses and the formulation of treatment plans.

The rest of this paper is organized as follows. Section 2 introduces the overall medical image segmentation and the detailed methods used for our spinal ultrasound image segmentation and reconstruction. The experimental settings and evaluation indicators are elaborated in Section 3. The experimental results are presented in Section 4. The discussion and conclusions are given in Sections 5 and Section 6, respectively.

## 2 Materials and methods

### 2.1 Overview

Image segmentation plays an important role in the quantitative and qualitative analyses of medical ultrasound images, and it directly affects subsequent analysis and processing (Patil and Deore, 2013). Correct segmentation guarantees the accurate extraction of diagnostic information from ultrasound images for clinical applications (Huang et al., 2021). It is also a crucial part of quantitative analysis in real-time clinical monitoring and precise positioning in computer-aided operations (Luo et al., 2021). To effectively visualize the 3D shape of the spine, we segmented the ultrasonic image. Medical ultrasound images have low image quality due to the limitation of imaging methods (Saini et al., 2010), but detailed features are an important basis for doctors’ diagnoses and identification. Therefore, the details of the original ultrasound image should be preserved as much as possible even though the ultrasound image is smoothed and denoised (Huang et al., 2020). To obtain detailed features and fine segmentation results, we need to derive global features and contextual information (Chen L et al., 2017). Previous studies have suggested enlarging the receptive field by expanding convolution and pooling operations (Chen C et al., 2017) or fusing mid- and high-level features with many task-related semantic features (Ronneberger et al., 2015; Zhao et al., 2017). However, these methods cannot capture contextual information in a global view and only consider the interdependencies between spatial domains.

In this work, we developed a new network structure called UGBNet. It uses an architecture based on the ResNet network module (He et al., 2016) to integrate features and unify the feature maps generated by each ResNet building block to the same size through interpolation. Concatenate and convolution operations are performed to achieve multi-scale feature fusion and generate multi-scale feature maps. We also incorporated a GGB module (Xue et al., 2021) that integrates spatial and channel attention through which long-range feature dependencies and contextual scale information are learned. Our UGBNet can integrate deep and shallow features to generate multi-level synthetic features as the spatial and channel-wise guiding information of non-local blocks (Chen L et al., 2017) and to complement the edge details that are usually ignored by deep CNNs. Guided by multi-level comprehensive features, our UGBNet can aggregate non-local features in spatial and channel domains, effectively combine long-term non-local features provided by distant pixels in ultrasound images, and learn the semantic information of powerful non-local features for an enhanced segmentation.

**FIGURE 1:**
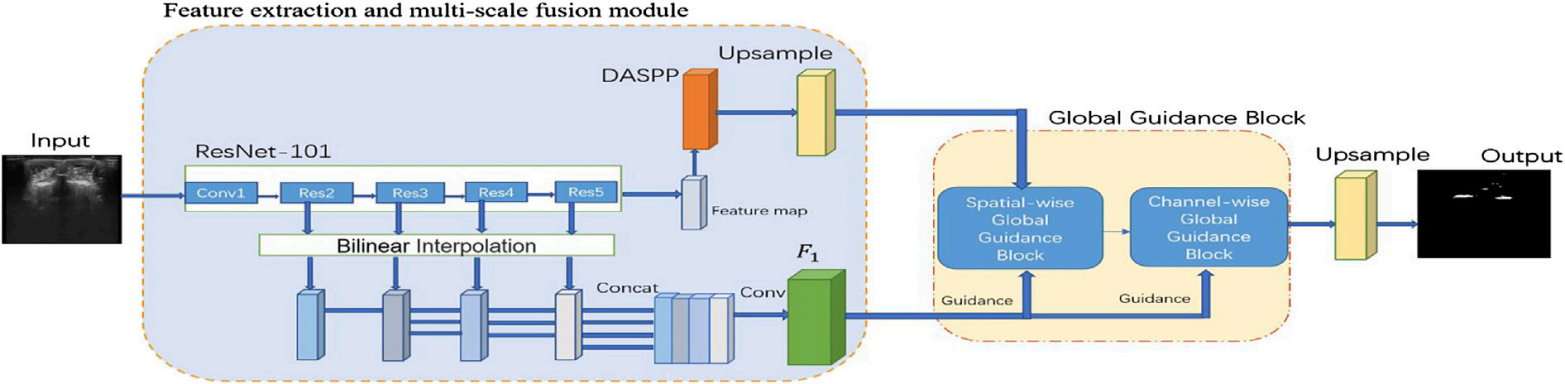
Schematic illustration of the proposed spinal ultrasound image segmentation network called UGBNet. ResNet-101 is used to extract dense local features.

Figure 1 shows the proposed UGBNet network structure. The network uses 2D spine ultrasound images as the input. First, Resnet’s structural blocks are employed to extract image features and then combined with the dense atrous spatial pyramid pooling (DASPP) module (Yang et al., 2018) to expand the receptive field. Second, the GGB module is introduced to make full use of the complementary information between different CNN layers. The GGB algorithm refines features by learning long-range feature dependencies under the guidance of low-level comprehensive feature maps. The output feature map of the GGB module is used as the prediction result of our network structure. After processing by the GGB module, the spatial-wise and channel-wise non-local features are aggregated to obtain an accurate segmentation effect.

### 2.2 Feature extraction and multi-scale fusion module

Image segmentation can be understood as a pixel-level classification problem. To identify the category that an image belongs to, we need to distinguish it from other image categories. Feature extraction plays an important role in image recognition and classification. Traditional feature extraction methods include scale-invariant feature transform and histogram of oriented gradient. With the development of deep learning, feature extraction through neural networks has been widely used. As one of the best approaches, ResNet has been widely adopted in image detection, segmentation, recognition, and other fields. ResNet designs a residual structure by using skip connection, which makes the network reach a deep level and endows it with an identity mapping ability and improved performance.

On the basis of the powerful feature extraction capability of ResNet, our UGBNet network structure adopts ResNet-101 (He et al., 2016) as the basic feature extraction network and uses 2D spine ultrasound images as the input. Each ResNet structure block can generate different feature maps to extract different features of the images. In particular, a DASPP module (Yang et al., 2018) is connected to the ResNet block to expand the receptive field, and its generated results are used as part of the input to the GGB module. The GGB module is discussed in Section 2.3.

To synthesize the semantic information of shallow and high-level networks, we need to carry out multi-scale feature fusion. Usually, different features can be observed at different scales to accomplish different tasks (Chen et al., 2016). With the deepening of the network layers, the receptive field of the network gradually enlarges, and the semantic expression ability is enhanced. However, this reduces the resolution of the image, and many detailed features become increasingly blurred after the convolutional operation of the multi-layer network. The convolutional neural network extracts the features of the target through layer-by-layer abstraction (Long et al., 2017). In the presence of only small local features or when the receptive field is too large, the obtained feature information is one-sided, and the possibility of obtaining too much invalid information arises. Using learned features at multiple scales helps encode global and local contexts.

In our paper, multi-scale feature prediction fusion is denoted as $F_{1}$ . We adopt ResNet-101 to extract dense local features. In this situation, because the features of each scale have different resolutions, they are up-sampled to a common resolution *via* bilinear interpolation. Then, feature maps from all scales are concatenated to form a tensor, which is convolved to create multi-scale feature prediction fusion. We generate feature maps from Res-2, Res-3, Res-4, and Res-5 in ResNet and unify them to the same scale through bilinear interpolation (Mastyło, 2013), channel stacking, and convolution to form a multi-scale fusion feature map $F_{1}$ , as illustrated in Figure 1. Therefore, our multi-scale fusion feature maps combine low-level details from shallow layers with high-level semantics learned in deep layers.

### 2.3 GGB

The traditional convolutional and recurrent operations of convolutional neural networks usually process only one local neighborhood and capture its spatial dependencies at a time (Wang et al., 2018). Although we can learn long-range dependencies by stacking convolutional layers, repeated local convolutions are time consuming. In addition, due to the limitation of imaging methods, spine ultrasound images usually contain speckles and shadows, and the signal-to-noise ratio is low (Bvsc, 2005). Diagnosis and identification pose difficulties. In this regard, we introduce GGB. The GGB module utilizes a guiding feature map to learn long-range dependencies by considering spatial and channel information, which is essential for achieving improved segmentation results.

#### 2.3.1 Spatial-wise GGB

**FIGURE 2:**
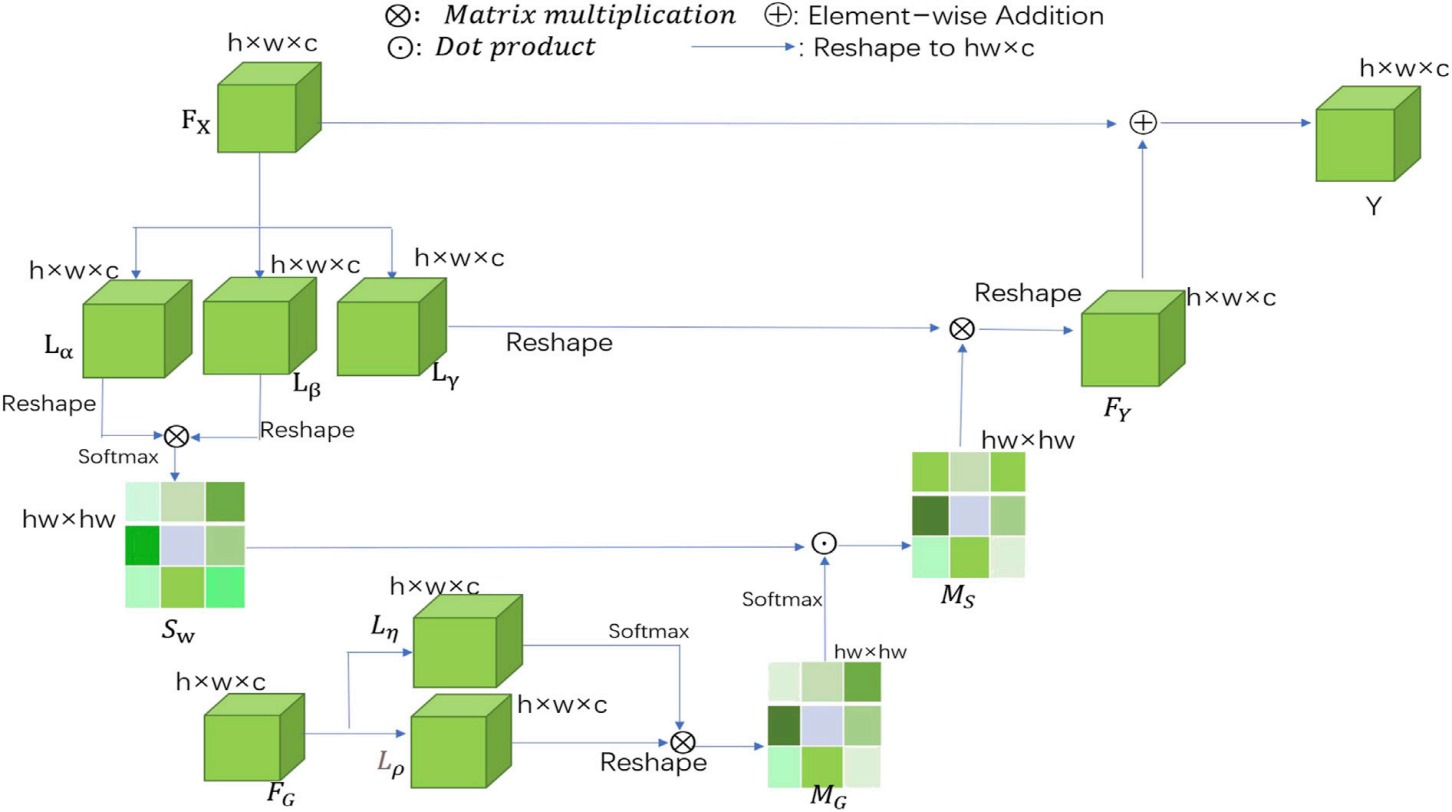
Schematic illustration of the details of spatial-wise GGB, where $F_{G}$ is the guidance map and $F_{X}$ is the input feature map.

Figure 2 presents our spatial-wise GGB module. The output feature map of the DASPP module (Yang et al., 2018) is represented by $F_{X}$ , and the guidance feature map is denoted as $F_{G}$ . $L_{\alpha \left(x\right)}$ , $L_{\beta \left(x\right)}$ , and ${L\;}_{\gamma \left(x\right)}$ are three 1×1 convolutional layers with different parameters, and $F_{X}$ is sent to them by the spatial-wise GGB module. Feature maps $\alpha_{\left(x\right)}$ , $\beta_{\left(x\right)}$ , and $\gamma_{\left(x\right)}$ are generated at the end. Then, matrix reshaping is performed. $\alpha_{\left(x\right)}$ , $\beta_{\left(x\right)}$ , and $\gamma_{\left(x\right)}$ are reshaped as $R^{hw\times c}$ matrices. In the end, we multiply the transpose of the reshaped $\alpha_{\left(x\right)}$ with the reshaped $\beta_{\left(x\right)}$ to derive a multiplication result. A softmax layer is applied to the multiplication result to calculate hw×hw spatial-wise position similarity map $S_{W}$ as follows:

$$S_{W}=Softmax\left(F_{X}^{T}L_{\alpha \left(x\right)}^{T}L_{\beta \left(x\right)}F_{X}\right).$$

The traditional sigmoid activation function is followed by a softmax layer, and it is applied to each $hw\times hwF_{X}^{T}L_{\alpha \left(x\right)}^{T}L_{\beta \left(x\right)}F_{X}$ . $L_{\eta \left(g\right)}$ and $L_{\rho \left(g\right)}$ are two 1×1 convolutional layers with parameters, and they are applied to guidance map $F_{G}$ . Afterward, we acquire feature maps η(x)and ρ(x), reshape η(x) and ρ(x), and multiply the reshaped η(x) to the transpose of the reshaped ρ(x). Then, a softmax layer is used again, which generates another hw×hw matrix of positional similarity from guidance map $F_{G}$ (denoted as $M_{G}$ ).

$$M_{G}=Softmax\left(F_{G}^{T}L_{\rho \left(g\right)}^{T}L_{\eta \left(g\right)}F_{G}\right))$$

When the two similarity matrices $S_{W}$ and $M_{G}$ are obtained, we conduct element-wise multiplication of $S_{W}$ and $M_{G}$ , and a softmax layer is applied to their result. This operation generates a guided similarity matrix $M_{S}$ . In the end, we multiply $M_{S}$ with the feature γ(x) to derive a new feature map $F_{Y}$ , which is then added with input feature $F_{X}$ to generate output feature map Y.

$$Y=\gamma \left(x\right)Softmax\left(S_{W}\cdot M_{G}\right)+F_{X}$$

#### 2.3.2 Channel-wise GGB

**FIGURE 3:**
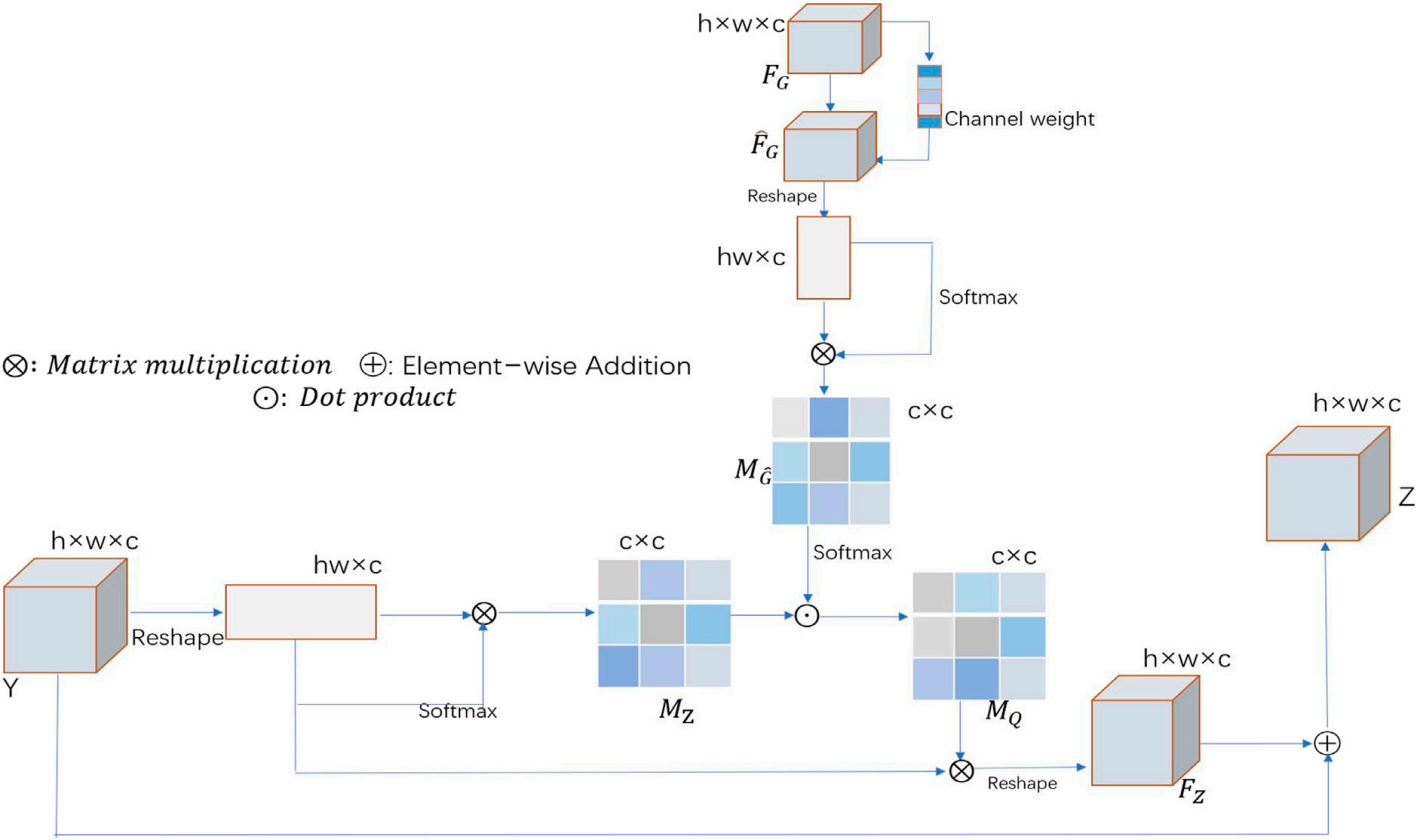
Schematic illustration of the channel-wise GGB, where $F_{G}$ is the guidance map and Y is the input feature map.

When learning long-range correlations, our spatial segmentation algorithm treats each feature channel equally and ignores the correlations between different feature channels. In recent years, many researchers have adopted strategies that allow for different contributions of different feature channels, thus achieving excellent results in many computer vision tasks (Hu et al., 2019; Lee et al., 2020). On this basis, a channel-wise GGB (channel-wise GGB) is introduced to further understand the long-range interdependencies among different feature channels. Figure 3 shows a schematic of the proposed channel-wise GGB. Feature map Y and guidance map $F_{G}$ are used as two inputs to the channel-wise GGB module. Refined feature map Z is subsequently generated. In addition, feature map Y is reshaped to $R^{c\times hw}$ ; we multiply the reshaped Y by its transpose and use the softmax layer to obtain channel-wise similarity feature map $M_{Z}\in R^{c\times c}$ . For input guidance feature map $F_{G}$ , the informative feature channels of channel $F_{G}$ are emphasized, and the less-used feature channel is suppressed using the squeeze-and-excitation block. For this purpose, we utilize global average pooling to generate channel-wise statistics λ, and the k-th layer element of the descriptor (λ) is given by

$$\lambda_{k}=\frac{1}{h\times w}\sum_{i=1}^{h}\;\sum_{j=1}^{w}\;F_{G}\left(i,j,k\right),$$

where $F_{G}\left(i,j,k\right)$ represents the element of the guidance map at position (i,j,k). Two fully connected (FC) layers and a sigmoid activation function are applied to channel-wise statistics λ, thus generating coefficient vector $V_{c}$ as follows:

$$V_{c}=\delta \left(P_{2}\phi \left(P_{1}\lambda \right)\right),$$

where $P_{1}$ and $P_{2}$ represent the parameters of the two FC layers and ϕ and δ are the ReLU and sigmoid activation functions, respectively. Next, we multiply $V_{c}$ with $F_{G}$ to assign different weights to the $F_{G}$ channel, resulting in a refined feature map (denoted as ${\hat{F}}_{G}$ ). After obtaining ${\hat{F}}_{G}$ , we reshape it to $R^{c\times hw}$ and multiply the reshaped ${\hat{F}}_{G}$ and the transpose of the reshaped ${\hat{F}}_{G}$ . A softmax layer is then used to generate c×c similarity feature map $M_{\hat{G}}$ . Subsequently, we multiply $M_{Z}$ with $M_{\hat{G}}$ , and a softmax layer is used for this process. At the end of this process, guided similarity map $M_{Q}$ is acquired. We multiply input Y with $M_{Q}$ to obtain new feature map $F_{Z}$ . $F_{Z}$ is added to input feature Y, which produces the output feature map Z of our channel-wise GGB.

### 2.4 Loss function

In this study, we use binary cross entropy (BCE) loss for network training. BCE is one of the widely used loss functions in two-class image segmentation tasks, and it reflects the direct difference between predicted masks and ground-truth labels. Its definition can be expressed as

$$\ell_{BCE}=-\underset{\left(i,j\right)}{\sum }\;Y\left(i,j\right)\cdot \log X\left(i,j\right)+\left(1-Y\left(i,j\right)\right)\cdot \log(1-X\left(i,j\right),$$

where Y(i,j)∈[0,1] represents the ground-truth label of pixel (i,j) and X(i,j)∈[0,1] denotes the predicted masks.

## 3 Experiments

### 3.1 Experimental settings

During training, we selected the ADAM optimizer to train our network. The initial learning rate of our network was 0.001. Multiple cross-validations showed that the segmentation performance was excellent when the epoch and batch sizes were set to 50 and 8, respectively. Our experimental device was a PC with four NVIDIA Geforce RTX 2080Ti GPUs. The development environment was Ubuntu 16.04, Python 3.6, and Pytorch 1.4.0. When outputting the training results in the testing phase, we used FC CRFs (Chen et al., 2014) on the refined segmentation results outputted by the GGB module to obtain the final predicted segmentation results. FC CRFs (Chen et al., 2014) can process the classification results obtained by deep learning in consideration of the relationship between all pixels in the image. It can also optimize the rough and uncertain labels in the classified image, correct the delicate misclassification areas, and obtain detailed segmentation boundaries.

### 3.2 Data acquisition

**FIGURE 4:**
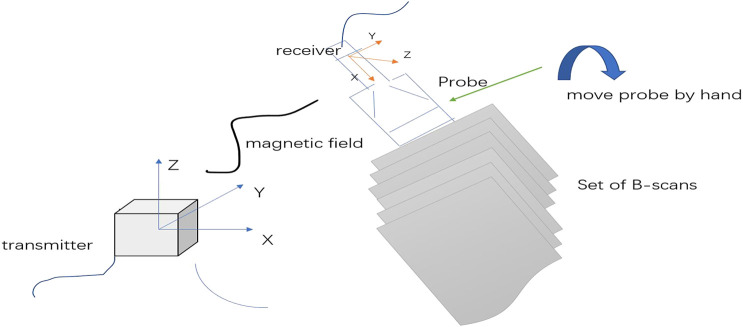
3D freehand ultrasound imaging. Freehand imaging allows the physician to move the probe freely so the B-scans can have arbitrary relative locations. The advantages of freehand scanning are low cost and scanning flexibility.

**FIGURE 5:**
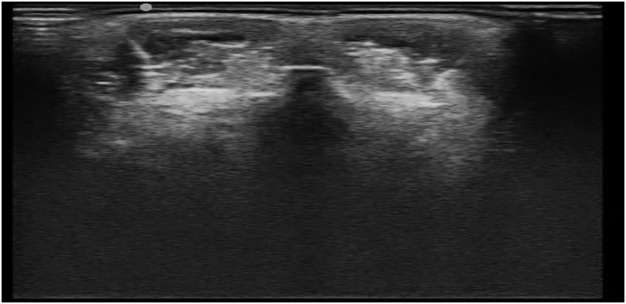
Typical spinal ultrasound image.

Our experimental data were scanned using the freehand 3D ultrasound imaging system (Figure 4 provides an illustration). Freehand 3D ultrasound refers to a 3D ultrasound formed by using traditional 2D black-and-white ultrasound diagnostic equipment combined with a certain positioning mechanism to obtain a series of 2D ultrasound images and the corresponding spatial positions through freehand scanning and perform 3D reconstruction (Gee et al., 2003). Freehand scanning is performed with a doctor’s hand-held probe, which is consistent with clinical ultrasound diagnosis and treatment applications, and the probe movement is not restricted. Images in any direction can be obtained. It is an economical, convenient, and flexible imaging method. Our study was conducted in accordance with local institutional review board standards, and all participants (or parents of participants under 18 years of age) provided written informed consent to participate in the study. A total of 102 AIS patients were recruited, and each participant could record approximately 2000 B-mode ultrasound images and their corresponding spatial data. The images we obtained were all 640×480 grayscale images, and the typical ultrasound images we used are shown in Figure 5.

### 3.3 Evaluation metrics

The similarity between the ground truth and CNN-based segmentation results can be assessed by employing several comparison metrics. We adopted four commonly used metrics to quantitatively compare different methods of spinal ultrasound image segmentation. The four metrics were Dice coefficient (denoted as Dice from hereon), Jaccard index (denoted as Jaccard from hereon), recall, and precision. Dice and Jaccard measure the similarity between the segmentation result and the ground truth. Precision and recall compute the pixel-wise classification accuracy to evaluate the segmentation result. In general, a good segmentation result has high values of these metrics. These evaluation metrics are calculated as follows:

1 Dice coefficient:

$$Dice\left(X,Y\right)=\frac{2\left|X\cap Y\right|}{\left|X\right|+\left|Y\right|}$$

2 Jaccard index:

$$\begin{array}{c} Jaccard\left(X,Y\right)=|intersection\;\left(X,Y\right)|/\left(\;union\;\left(X,Y\right)\right)\\ =\frac{\left|X⋂Y\right|}{\left|X⋃Y\right|}\\ =\frac{\left|X⋂Y\right|}{\left|X\right|+\left|Y\right|-\left|X⋂Y\right|} \end{array}$$

where X is the gold standard, which is the average result marked by experienced clinical experts; Y is the region segmented by the model; and X∩Y represents the region of overlap between the gold standard and the segmentation output of the model.

3 Recall:

$$Recall=\frac{TP}{TP+FN}$$

4 Precision:

$$Precision=\frac{TP}{TP+FP}$$

The calculation of recall and precision is associated with the true positive (TP), true negative (TN), false positive (FP), and false negative (FN) of the confusion matrix. TP is the positive image block correctly recognized by the network. FP is a negative image block that is incorrectly identified by the network as belonging to the positive image block. FN is a positive image block that is not recognized as belonging to the target image block.

## 4 Results

### 4.1 Segmentation results

To evaluate the segmentation effect of different network structures, we performed ablation experiments on our dataset. To accomplish the task of cross-validation on the dataset, we also conducted data labeling (the training dataset was labeled). We invited relevant practitioners to serve as a guide in annotating bony features, such as spinous processes, transverse processes, and ribs, in the 2D images obtained by our ultrasound scan; the features were then used as the ground truth in our experimental dataset. We compared our network against several deep-learning-based segmentation methods, including Unet (Ronneberger et al., 2015), NestedUnet (Zhou et al., 2018), SegNet (Badrinarayanan et al., 2017), and CENet (Gu et al., 2019). To ensure the fairness of the comparison, all comparative experiments were performed on the same spinal ultrasound dataset *via* four-fold cross-validation.

**FIGURE 6:**
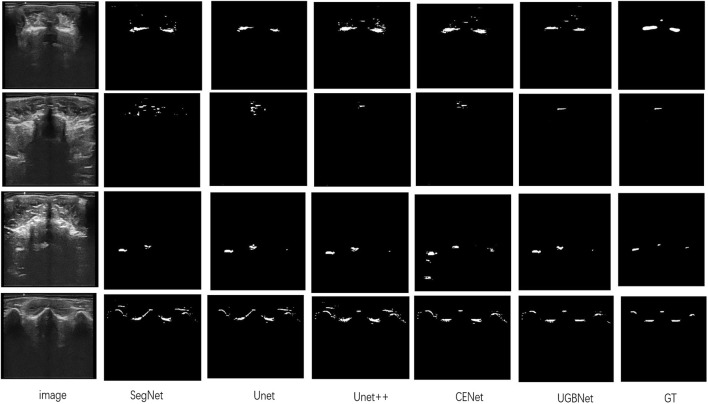
Segmentation results of different methods on our spinal ultrasound image dataset. The ground truth is denoted as GT. From left to right are our original images and the segmentation results of SegNet, Unet, Unet++, CENet, and our method. The last column is the ground truth.

Visual comparison. According to the visualization results of the segmentation shown in Figure 6, our approach precisely segmented the spinous processes and laminae from the ultrasound images despite the presence of serious artifacts, whereas the other methods tended to generate over- or under-segmented results. Our network could successfully segment images with vague boundaries and detect small objects in the images. Its results were the most consistent with the ground truth among all the segmentation results.

Quantitative comparison. The quantitative evaluation of the segmentation results of spinal ultrasound images produced by the different segmentation methods is presented in Table 1. Compared with the other methods, our approach achieved higher values on Dice, Jaccard, precision, and recall measurements, demonstrating the high accuracy of the proposed approach in spinal ultrasound image segmentation.

**TABLE 1:** Quantitative evaluation of different methods for spinal ultrasound image segmentation.

	Dice%	Recall%	Jaccard%	Precision%
UGBNet	74.2 ± 1.2	78.5 ± 1.8	66.8 ± 1.5	79.5 ± 1.6
Unet	63.3 ± 1.6	62.2 ± 2.1	56.8 ± 1.4	68.4 ± 1.5
SegNet	61.3 ± 1.6	64.3 ± 1.8	54.3 ± 1.2	65.2 ± 1.4
Unet++	68.8 ± 1.3	71.2 ± 1.4	58.9 ± 1.6	73.5 ± 2.1
CENet	71.5 ± 1.4	75.1 ± 1.6	59.5 ± 1.7	77.2 ± 1.1

## 5 Discussion

At present, the clinical measurement of scoliosis is mainly based on X-rays, but the radiation of X-rays makes it difficult to be used for long-term monitoring (Kim et al., 2010). Compared with X-rays, the new spinal ultrasound imaging method is a real-time, economical, radiation-free technology (Ahmed et al., 2018). However, ultrasound images also have their inherent limitations. Given the limitations of imaging methods, ultrasound images often have acoustic artifacts, spots, and reticulated noise, which easily hide bony features, such as spinous and transverse processes, thereby making manual recognition and segmentation increasingly difficult. Inspired by Ungi et al., our research group adopted a two-stage processing strategy for the measurement and visualization of scoliosis, that is, the spine ultrasound image was segmented and recognized, the irrelevant information and noise were eliminated, and 3D visualization of spine shape was carried out. The main contribution of our study is the development of a novel segmentation network structure called UGBNet for spine ultrasound images; UGBNet performs feature extraction and multiscale fusion and incorporates a GGB module, which learns long-range feature dependencies, aggregates non-local features in spatial and channel domains, and refines the features to obtain accurate segmentation results.

Traditional spine imaging often shows the spine morphology through 3D reconstruction, which is performed directly using images obtained from ultrasound sweeps (Cheung et al., 2015). However, due to the limitations of the depth setting of the ultrasonic probe and the surrounding magnetic field, the quality of the captured 2D images cannot be guaranteed. The large amount of shadows and noise in low-quality images bring difficulties to the subsequent image recognition and 3D reconstruction visualization. This study proposed a two-stage processing strategy, that is, the bony features in the spine ultrasound image are recognized and segmented, followed by 3D reconstruction and visualization of the spine. This two-stage processing strategy can minimize the interference of irrelevant information, such as acoustic artifacts and speckle noise, and has positive significance for the subsequent visualization of 3D spine morphology and measurement of scoliosis degree. In the segmentation and recognition of bony features of spine ultrasound images, we improved the segmentation algorithm and proposed the UGBNet network structure. Multiple qualitative and quantitative experiments showed that our method achieved higher values of Dice, precision, and other evaluation metrics compared with traditional image segmentation algorithms, such as UNet.

However, our method cannot guarantee accurate segmentation of all spinal ultrasound images. In terms of ultrasonic data acquisition, due to the inexperience of the operator, some scanning problems may arise, resulting in the poor quality of the collected 2D ultrasonic images and the presence of abundant shadows and noise (Rohling et al., 1999). Our methods are often inadequate when dealing with such images. In our future research, we will consider preprocessing the acquired 2D ultrasound image to enhance the weight of the structure of interest, find ways to improve the image contrast and image quality, and lay a good foundation for subsequent research. In addition, the performance of deep learning networks needs to be tested in a larger and more diverse patient population than the current one (Cai et al., 2020). Our sample size is relatively small, and continued large-scale clinical trials are needed to validate the feasibility of using the proposed method in the diagnosis, treatment, and screening of scoliosis.

## 6 Conclusion

In summary, we propose a novel spinal ultrasound image segmentation network called UGBNet, which can accurately segment and identify bony features, such as spinous and transverse processes, in spinal ultrasound images. The proposed network considers long-range dependencies in a spatial-wise and channel-wise manner and embeds contextual information from different layers. Our method can be used as the first step in a two-stage processing strategy for spinal ultrasound 3D imaging and scoliosis measurement, which is important for subsequent visualization of spinal 3D morphology and scoliosis measurement. Our approach is radiation-free and inexpensive, and it provides a new idea for the clinical measurement and treatment of scoliosis. It is a feasible alternative to current approaches that use X-ray as the main diagnostic method, and we look forward to its large-scale promotion in the future.

## Data Availability

The raw data supporting the conclusion of this article will be made available by the authors, without undue reservation.
